# Survey dataset on Muslim’s religiosity, Muslim personality and work behavior

**DOI:** 10.1016/j.dib.2018.10.169

**Published:** 2018-11-03

**Authors:** Ahmad Amri Zainal Adnan, Naveed R. Khan, Siti Asma binti Rosdi, Nek Kamal Yeop Yunus, Arsalan Mujahid Ghouri, Mirza A. Haq

**Affiliations:** a Universiti Pendidikan Sultan Idris, Malaysia; b Bahria University, Pakistan; c Iqra University, Pakistan

## Abstract

Data were collected from administration officers ranging from middle-management to top management of the five universities of Malaysia. The data was collected through a standardized and structured questionnaire. The variables of the study were religiosity, personality and work behavior of Muslims. Muslim work behavior construct formulated on the basis on collected data.

**Specifications table**

**Table:** 

Subject area	Organizational Behavior, Psychology
More specific subject area	Psychology of Religion
Type of data	Table and text file
How data was acquired	Survey, SEM,
Data format	Analyzed, descriptive and statistical data
Experimental factors	Religiosity, personality and work behavior gauged in the study. A 20-items religiosity scale, 60-items Personality scale were adopted from existing studies and 37-items Muslim work behavior newly developed in current study.
Experimental features	Data were gained through questionnaires using stratified random sampling. Questionnaires were screened manually for missing values or irrelevant values before the data analysis. KMO, Bartlett, and Reliability applied before confirmatory factor analysis.
Data source location	Public University in Selangor and Negeri Sembilan, Malaysia.
Data accessibility	All the data are in this data article as a supplementary data file
Related research article	Brotheridge, C. M. & Lee, R.T. 2007. Hands to work, heart to God: religiosity and organizational behavior. Journal of Management, Spirituality & Religion 4 (3): 287-309.
	Saroglou, V. 2002. Religion and the five factors of personality: A meta-analytic review. Personality and Individual Differences 32: 15-25.

**Value of the data**

• It is the premier data collection on Muslim work behavior construct build by Adnan (2016).
• The data will be useful for an analysis in various areas, that is, Psychology and Organizational Behavior, that is, organizational psychology, developmental psychology, cultural impact, norms and beliefs and behavior, religion as actor of change.
• The study can be replicated to other countries with similar demographic factors.
• The results can be use to determine how to use religiosity to change behavior in the workplace.
• The results can give an insight on Muslim work behavior constructs.
• The assessment tools can be use in similar study.

## 1 Data

### 1.1 Demographic characteristics of respondents

Data were collected from administration officers ranging from middle-management to top management of the five universities of Malaysia chosen for this research. These universities are top five public sector universities by number of students. Of these members, the final sample used in this study consisted of 228 members, who provided complete information for the three constructs in the survey. Data collection adhere all ethical consideration suggested by prominent studies [1], [2], [3]. Of the 228 members constituted the final sample, 42% were male, 57% were female and 1% did not stated gender; and age ranged from 23 to 60 years old. In terms of age groups, the majority 34.2% were 30–35 years old. All respondents were followers of Islam. The Table 1 illustrates the details.

**Table 1:** Demographics.

Name	Type	Frequency	Percent
Gender	Male	96	42.1
	Female	130	57.0
	Missing value	2	0.9
Age	<30	44	19.3
	30–35	78	34.2
	36–40	46	20.2
	41–45	23	10.1
	46–50	15	6.6
	51 and above	20	8.8
	Missing value	2	0.9

## 2 Experimental design, materials, and methods

### 2.1 Research instrument

A survey design was used to collect data on religiosity, personality and work behavior. Religiosity, defined as the individual’s beliefs and understanding towards elements that shape a certain religion. A 20-items religiosity scale (MRPI) was adapted from Krauss et al. [4]. Personality scale (NEO) was measured with a 60-items scale developed and validated by Costa et al. [5]. However, Muslim work behavior (MWBI) was measured with a 37-items scale newly developed by Adnan [6].

The Religiosity is measured through the manifestation of Islam: faith and courtesy. Muslim religiosity consists of two main constructs: Islamic Worldview (Tasawwur Islam) and Religious Personality. However, the Religious Personality was dropped from analysis due to items overlap with MWBI (discuss later). Construct of Islamic Worldview is a paradigm of belief (tawhid) and understanding (aqidah) of the six pillars of faith in Islam. The Islamic Worldview construct consists of two sub-constructs, namely Worldly and Spiritual which consists of 10 items each. Worldly sub-construct assess the level of belief and understanding of the linkage of Islam with life in the world, while Spiritual sub-construct assesses the beliefs and understanding of God׳s relationship with other creatures and elements.

The Personality dimension measured by NEO Five-Factor Inventory (NEO FFI). NEO FFI is a short version of NEO Personality Inventory-Revised (NEO PI-R) developed by Costa et al. [5]. Each trait in NEO FFI is represented by 12 items. Therefore, NEO FFI consists of 60 items to measure five personality traits in the big five or five factor model, namely Neuroticism, Extraversion, Openness to Experience, Agreeableness and Conscientiousness.

MWBI measures the behavior of Muslims through two constructs: Ability (al-Qawiy) and Trustworthiness (al- Amīn). The main construct of the instrument was formed from the verse of the Quran (Al-Qas.as. 28: 26) which stated that Ability and Trustworthiness are the two characteristics of an excellent worker. Sub-construct were then formed based on other related verses of the Quran, hadith, ideas from Muslim clerics and thinkers. An interview with a subject matter expert in human resource management was also done to enhance the reliability of the sub-constructs. Overall 24 sub-constructs were identified (Ability = 15, Trustworthiness = 9,). In the end of analyses, Constructs of Ability include three sub-constructs: Professional, Competent and Sensitivity; while Trustworthiness constitutes sub-constructs of Integrity, Fairness and Principled. Trustworthiness tool has 37 items.

### 2.2 Details of MWBI construct building

#### 2.2.1 KMO, Bartlett, and reliability

In the start, 51 items were formed from 24 sub-constructs, with reference to the Quran and hadiths. The items were later shown to experts to be assess and validated. Pilot study of the 51 items was done with 100 respondents with the same characteristics as the targeted population. Authors ran a factor analysis with principal component factoring [7] and varimax rotation [8] separately for each construct. In order to determine whether the factor analysis was appropriate for the data set, we checked the Kaiser–Meyer–Olkin (KMO) [9] measure of sampling adequacy and Bartlett’s test of sphericity [10].The KMO for both constructs was above 0.500 (Ability = 0.910, Trustworthiness =;0.909), suggesting that the data was suitable for factor analysis. Moreover, Bartlett’s test resulted in a highly significant chi-square statistic for Ability (*χ*^2^ = 2133, *p *= 0.000) and Trustworthiness (*χ*^2^ = 1,441, *p* = 0.000), indicating adequate correlation among the items. Thus, factor analysis was appropriate for the existing data set. The factor analysis of Ability showed that three sub-constructs: Professional, Competency and Sensitivity together explained 59.9% of the total variance. While factor analysis for Trustworthiness showed three sub-constructs: Integrity, Fairness and Principled together explained 61.7% of the total variance. Construct validity, determined through the presence of convergent and discriminant validity, demonstrates how well the measurement items relate to the constructs.

To demonstrate convergent validity [11], we use two tests: item reliability [12] and average variance extracted (AVE) [13]. We determined item reliability by examining construct item loadings. In general, loadings at or above 0.50 demonstrate adequate item reliability. All the Cronbach alpha values are between 0.82 and 0.93. Finally, the average variance extracted (AVE) represents the amount of variance a construct captures via its items relative to the amount of variation due to measurement error. We found that each construct’s variance extracted was above the recommended value of 0.5. Thus, it concluded that all our constructs had satisfactory convergent validity [14]. For discriminant validity test, a construct’s variance extracted, or shared variance between the construct and its items, should be greater than the shared variance between the construct and other constructs; this was measured by comparing the square root of a construct’s average variance extracted (AVE) to its correlations with other constructs. For each construct, authors observe that the square root of the AVE is considerably larger than its correlations with other constructs. Consequently, our constructs demonstrate adequate discriminant validity.

Two items were dismissed from Ability construct because the cross-loading between sub-constructs are >0.50. Cronbach reliability test of the construct after the two items were remove shows a value of 0.96. For Trustworthiness, one item was dismissed from the construct because the cross-loading value is >0.5. Cronbach alpha reliability test after the item was dismissed shows a value of 0.94. Overall, 48 items obtained after the exploratory factor analysis with three sub-constructs each of Ability and Trustworthiness.

#### 2.2.2 Results of confirmatory factor analysis

By using AMOS software, confirmatory factor analysis (CFA) is carried out to test whether the construct measurement is consistent with the theory presented to obtain the validity of the constructs. At the measurement model stage, the validation analysis of each latent construct is done individually for first order CFA.

##### 2.2.2.1 Results of first order measurement model

The results the fitness of all sub-constructs are presented in Table 2. For the Professional sub-construct, regression weighting of all 14 items were found to be >0.60 and the value of all fitness index showed that the fitness of this sub-construct model was achieved. All 14 items were retained in this sub-construct. The regression weighted value of all eight Competitive sub-construct items were >0.60 and the value of all fitness index shows that the fitness of this sub-construct model has been achieved. Therefore, all eight items were retained in this sub-construct. For the Sensitivity sub-construct, only four (five) regression weighted values were found to be >0.60 and one item had to be removed from sub-construct. The value of all fitness index indicates that the fitness of this sub-construct model has been achieved. Hence, four items were retained in this sub-construct.

**Table 2:** Compatibility test results.

Test category/construct	Absolute fit		Incremental fit	Parsimonious fit
	RMSEA	GFI	CFI	Chi sq/df
Professional	0.078	0.901	0.943	2.399
Competency	0.084	0.952	0.959	2.601
Sensitivity	0.068	0.991	0.993	2.044
Integrity	0.096	0.963	0.953	3.079
Fairness	0.081	0.978	0.976	2.479
Principled	*0.000*	–	0.994	0.569
Ability	0.082	*0.827*	0.900	2.509
Trustworthiness	0.086	0.905	0.924	2.672
MWBI	0.064	0.982	0.995	1.940

The integrity sub-construct, only six of the eight sub-construct items had the regression weight value of >0.60.The value of all fitness index indicates that the fitness of this sub-construct model has been achieved. Therefore, six items were retained in this sub-construct. For the Fairness sub-construct, only five out of eight items had regression weight value >0.60. The value of all fitness index indicates that the compatibility of this sub-construct model has been achieved. Apparently, five items were retained in this sub-construct. For Principled sub-construct, the regression weight value of four of the five items is >0.60. After an item was discarded, all fitness index values show that the fitness of this sub-construct model has been achieved except for the RMSEA index, where it is <0.05, which according to [15] is the minimum value of the RMSEA index. However, absolute fit has been achieved through the Goodness of Fit (GFI) index. Thus, four items were retained in this sub-construct. Last first-stage measurement model, it was found that one item of the Ability construct and six items of the Trustworthiness construct had to be eliminated to obtain the corresponding model of the two constructs. Overall, one item removed from Ability sub-construct and six from Trustworthiness sub-construct. Total seven items were removed from the list of 48 items of MWBI. A total of 41 items (Ability = 26, Trustworthiness = 15) were subsequently analyzed to the second level.

Two of the 26 Ability sub-construct items were eliminated in the second stage of the confirmatory factor analysis because the regression weight value was <0.60. After two items were omitted, the value of all fitness index indicates that the fitness of this sub-construct model has been achieved except for the GFI index where it is <0.90 [16]. However, absolute fit is achieved through the RMSEA. Additionally, two of the 15 items in the Trustworthiness sub-construct had to be removed, had regression weight value of <0.60. After the two items were omitted, all fitness index values indicate that the fitness of this sub-construct model has been achieved. At this stage, total 37 items were retained for MWBI construct.

The results of the confirmatory factor analysis, with the Maximum Likelihood Estimates, show that the critical ratio of the regression coefficient between Ability latent variables with the three variables of the indicator (Professional, Competent, and Sensitivity) as well as Trustworthiness pending variables with all three indicators (Integrity, Fairness, and Principled) are outside the range of ±1.96, and the standard regression coefficient (β) for each path between latent variables and its indicator variables is between 0.78 and 0.94. The results of this analysis show that both the Ability and Trustworthiness latent variables can be represented by their respective indicator variables.

The fitness test of the confirmatory factor analysis model found that all fitness index values, Root Mean Square of Error Approximation (RMSEA), Goodness of Fit Index (GFI), Comparative Fit Index (CFI), and Chi Square / Degree of Freedom (Chisq/df) used demonstrate fitness has been achieved [17], [18], [19], [20], [21], [22]. The results of the model fitness test are shown in Table 2.

The Squared Multiple Correlation results show that 0.61–0.89 (61%–88.9%) variance in the indicators variables in the equation model can be predicted by the research data. Table 3 shows the reliability and validity of the constructs in MWBI after the confirmatory factor analysis.

**Table 3:** Reliability and validity of MWBI constructs.

Construct	Cronbach alpha	Reliability of constructs	Average variance extracted (AVE)
Ability	0.956	0.962	1.766
Trustworthiness	0.909	0.911	0.820

The Table 4 explains the square root value of Average Variance Extracted (AVE) of Ability (1.33) and Trustworthiness (0.91) is greater than the correlation between Ability and Trustworthiness (0.87), then discriminant validity has been achieved.

**Table 4:** Discriminant validity index.

	**Ability**	**Trustworthiness**
Ability	1.33	
Trustworthiness	0.87	0.91
