# Supplementary material for: Survey dataset on Muslim’s religiosity, Muslim personality and work behavior
Source: Data Brief. 2018 Nov 3;21:1880–5. doi: 10.1016/j.dib.2018.10.169 (PMC6260372; doi:10.1016/j.dib.2018.10.169)
Supplement: Supplementary file 1 — Supplementary material [file mmc1.doc]

To,

Editor-in-Chiefs,

Data in Brief,

07^th^ October 2018

Dear Dr. Wand and Dr. Lan,

I’m pleased to submit an original article’s revised version entitled ‘**Survey dataset on Muslim’s Religiosity, Muslim Personality and Work Behavior**’ by Ahmad Amri Zainal Adnan, Naveed R Khan, Amsa binti Mohd Rosli, Nek Kamal Yeop Yunus, Arsalan Mujahid Ghouri, and Mirza A Haq for consideration for publication in ‘esteem **Data in Brief.** We confirm that this work is original and same has not been published elsewhere, nor is it currently under consideration for publication elsewhere.

In this paper, authors studied the religiosity, personality and work behavior of Muslims. Muslim work behavior construct formulated on the basis on collected data.

I believe that this manuscript is appropriate for publication in **Data in Brief**.

We have no conflicts of interest to disclose.

Thank you for your consideration of this manuscript.

Sincerely,

Arsalan Mujahid Ghouri, PhD
Senior Lecturer,

Universiti Pendidikan Sultan Idris,

Malaysia.

Email: [arsalan@fpe.upsi.edu.my](mailto:arsalan@fpe.upsi.edu.my) , [arsalan.ghouri@ymail.com](mailto:arsalan.ghouri@ymail.com)

Contact: +60142728233
